# Mapping of secondary forest age in China using stacked generalization and Landsat time series

**DOI:** 10.1038/s41597-024-03133-2

**Published:** 2024-03-16

**Authors:** Shaoyu Zhang, Hanzeyu Xu, Aixia Liu, Shuhua Qi, Bisong Hu, Min Huang, Jin Luo

**Affiliations:** 1 Key Laboratory of Poyang Lake Wetland and Watershed Research (Ministry of Education), School of Geography and Environment, Jiangxi Normal University, Nanchang, 330022 China; 2 School of Geography, Nanjing Normal University, Nanjing, 210023 China; 3 Land Satellite Remote Sensing Application Center, Ministry of Natural Resources, Beijing, 10048 China

**Keywords:** Forestry, Forest ecology

## Abstract

A national distribution of secondary forest age (SFA) is essential for understanding the forest ecosystem and carbon stock in China. While past studies have mainly used various change detection algorithms to detect forest disturbance, which cannot adequately characterize the entire forest landscape. This study developed a data-driven approach for improving performances of the Vegetation Change Tracker (VCT) and Continuous Change Detection and Classification (CCDC) algorithms for detecting the establishment of forest stands. An ensemble method for mapping national-scale SFA by determining the establishment time of secondary forest stands using change detection algorithms and dense Landsat time series was proposed. A dataset of national secondary forest age for China (SFAC) for 1 to 34 and with a 30-m spatial resolution was produced from the optimal ensemble model. This dataset provides national, continuous spatial SFA information and can improve understanding of secondary forests and the estimation of forest carbon storage in China.

## Background & Summary

Secondary forests represent forest or wood ecosystems that have recovered from disturbance following regeneration or plantation^1^. Secondary forests dominate the forest landscape and play a crucial role in ecosystem health^2^. Therefore, understanding the structure characteristics of secondary forests is important for developing forest conservation policies^3^. For example, stand age is an indicator of the forest ecosystem with ecological relevance^4–6^. However, few past studies on secondary forests have less interest in stand age since the main focuses have been on the extent and range of forest disturbance. Therefore, there is a need to estimate secondary forest age (SFA) to improve understanding of the function of secondary forests in the national-scale terrestrial ecosystem^7^.

Remote sensing technologies offer low-cost, efficient, and easily accessible data at multiple spatial and temporal scales, and these data provide exciting possibilities for investigating the resources, composition, and functions of forests^8^. There are currently three typical categories of methods for mapping SFA: (1) derivation from the land cover datasets; (2) classification from remote-sensed images; and (3) retrieving the establishment time of secondary forest stands. The choice of derivation method is heavily dependent on the accuracy and period of continuous land cover datasets, and these datasets can be difficult to obtain at a large scale^1,9,10^. Classification-based SFA mapping offers the opportunity to differentiate between mature and secondary forests. This approach typically uses high-resolution satellite imagery, including SPOT-5, WorldView-3, and ALOS PALSAR^11–13^. Time series data of land disturbance can be utilized for mapping the age of tree crops (e.g., oil palm, rubber) based on change detection algorithms^14–16^. Therefore, historical SFA can be estimated by retrieving the establishment time of the secondary forest using time series change detection algorithms.

Many time series change detection algorithms have been proposed for analyzing the historical dynamics of forests^17–19^. Some previous estimates of SFA by monitoring forest stands after disturbance at regional scales^1,20–23^ using time series segment algorithms, such as the Vegetation Change Tracker (VCT)^24,25^ and Continuous Change Detection and Classification (CCDC)^26^. However, the use of a single approach to retrieve the time of stand establishment is inadequate due to the complexity of the terrain, the range of forest types, and the basic algorithm logic. There has been limited focus and progress on the recovery of the secondary forest age since most algorithms have been designed for detecting forest disturbance^27^. In addition, the application of these algorithms to different regions has highlighted their continued inconvenience and uncertainty. For example, the widely-used VCT remains difficult to apply at large scales and the CCDC continues to overestimate change due to its sensitivity to subtle changes^28^. Thus, there remains a need to improve the understanding and accuracy of change detection algorithms to allow the precise mapping of regional- and national-scale SFA.

Forest accounts for 23.04% of the total area of China (2021) (http://www.forestry.gov.cn/). This high coverage of forest in China can be mainly attributed to afforestation and forest recovery efforts over the past decades, including programs to return cultivated land into forest and the closing hillsides to facilitate afforestation. Estimations of the distribution, density, structure, and pattern of secondary forests are key for understanding the role and function of secondary forests within the wider forest ecosystem in China. The aims of the present study were to (1) develop a data-driven method for VCT and CCDC on detecting the establishment of forest stands; (2) design a novel SFA estimation method using stacked generalization and Landsat time series; (3) use the optimal ensemble method to produce a national-scale mapping of SFA for China for 2020; (4) assess the accuracy of SFAC^29^ with validation samples, statistical data, and other datasets.

## Methods

### Data processing

#### Landsat time series data

The present study used Landsat Collection 2 Level-1 data to retrieve the establishment time of secondary forest stands. All available surface reflectance images, including Landsat 5 Thematic Mapper (5TM), 7 Enhanced Thematic Mapper Plus (7ETM+), and 8 Operational Land Imager (8OLI) images from 1986 to 2021 were provided by the United States Geological Survey (USGS) (available at https://earthexplorer.usgs.gov/) and were obtained using Google Earth Engine (GEE)^30^. Clouds, cloud shadows, and snow were filtered using CFMask algorithms^31^ in GEE. The stacks of the annual composite were obtained using the Best Available Pixel (BAP) method^32^ and were used as input data for the LandTrendr (LT) and VCT algorithms described below. The stacks of all available images were prepared as inputs for the Moving Average Change Detection (MACD) and CCDC algorithms described below.

#### New reference forest map

**Fig. 1:**
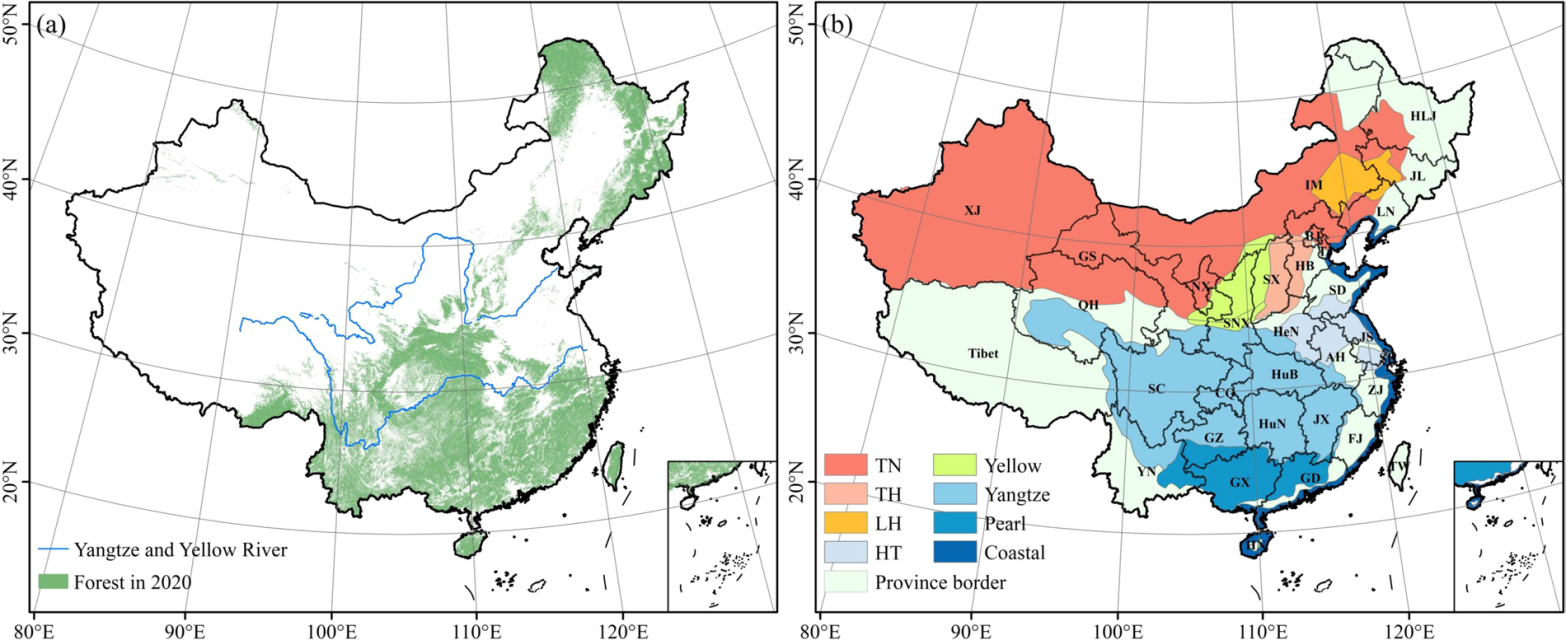
The new forest map in 2020 and eight forestry projects and provinces in China. (**a**) The new forest map for China in 2020 and (**b**) the distribution of eight forestry projects.

The present study produced a new reference forest map for China from three land cover products for 2020^33^ (Fig. 1a). These datasets included the World Cover 2020 (ESA-2020) (available at https://esa-worldcover.org/en), ESRI 2020 Land Cover (ESRI-2020) (available at https://livingatlas.arcgis.com/landcover), and the GlobeLand30 version of V2020 (GLC-2020) (available at http://www.globallandcover.com/). The ESA-2020 dataset is a global land cover map with an overall accuracy for Asia 2020 of 80.7% at a 10-m resolution^34^. The ESRI-2020 co-released by ESRI and Microsoft Planetary Computer platform is a global land cover map with a 10-m resolution^35^. GLC-2020 was created by a research group in China and is a global land cover product with a 30-m resolution and an overall accuracy for 2020 of 85.72%^36,37^. The “forest” category is all provided in these land cover products, though from inconsistent definitions (i.e., tree cover percentage >15% and tree height >3 m in ESA-2020, vegetation cover with trees >30% in GLC-2020). Within the production of the reference forest map for China, a pixel was assumed to represent forest when the same pixel in at least two land cover products showed forest properties, thereby decreasing the uncertainties of classification of forests at a large scale^38,39^.

Figure 1 shows the distribution of the forest baseline map and the multiple sub-study districts in China. These sub-study districts include eight forestry projects and 31 provinces: the three-north shelterbelt program (TN); afforestation program for Taihang Mountain (TH); shelterbelt program for Liaohe river (LH); shelterbelt program for the middle reaches of the Yellow river (Yellow); the shelterbelt program for Huaihe river and Taihu lake (HT); the shelterbelt program for the upper and middle reaches of the Yangtze river (Yangtze); the shelterbelt program for the Pearl river (Pearl); the coastal shelterbelt program (Coastal). The provinces of China and adjacent areas included in the present study are Anhui (AH), Beijing (BJ), Chongqing (CQ), Fujian (FJ), Guangdong (GD), Gansu (GS), Guangxi (GX), Guizhou (GZ), Hebei (HB), Henan (HeN), Heilongjiang (HLJ), Hainan (HN), Hubei (HuB), Hunan (HuN), Inner Mongolia (IM), Jilin (JL), Jiangsu (JS), Jiangxi (JX), Liaoning (LN), Ningxia (NX), Qinghai (QH), Sichuan (SC), Shandong (SD), Shanghai (SH), Shannxi (SNX), Shanxi (SX), Tibet, Tianjin (TJ), Taiwan (TW), Xinjiang (XJ), Yunnan (YN), Zhejiang (ZJ).

#### Candidate stable and secondary forest maps

Candidate stable and secondary forest maps were prepared for validation against samples and input into the algorithms. The European Space Agency Climate Change Initiative-Land Cover (ESA_CCI-LC) project provides a consistent annual global land cover map with a 300-m spatial resolution for 1992 to 2020^40^ (available at https://climate.esa.int/en/projects/land-cover/). The stable and secondary forest maps were individually derived based on ESA_CCI-LC by yearly overlaying^10^. Pixels of stable forest represented the forest in 1986 was always there from 1986 to 2020 without clear-cut or regrowth, whereas pixels of secondary forest were identified as the newly occurred forest including the natural forest regrowth and artificial afforestation.

#### Validation Samples

**Fig. 2:**
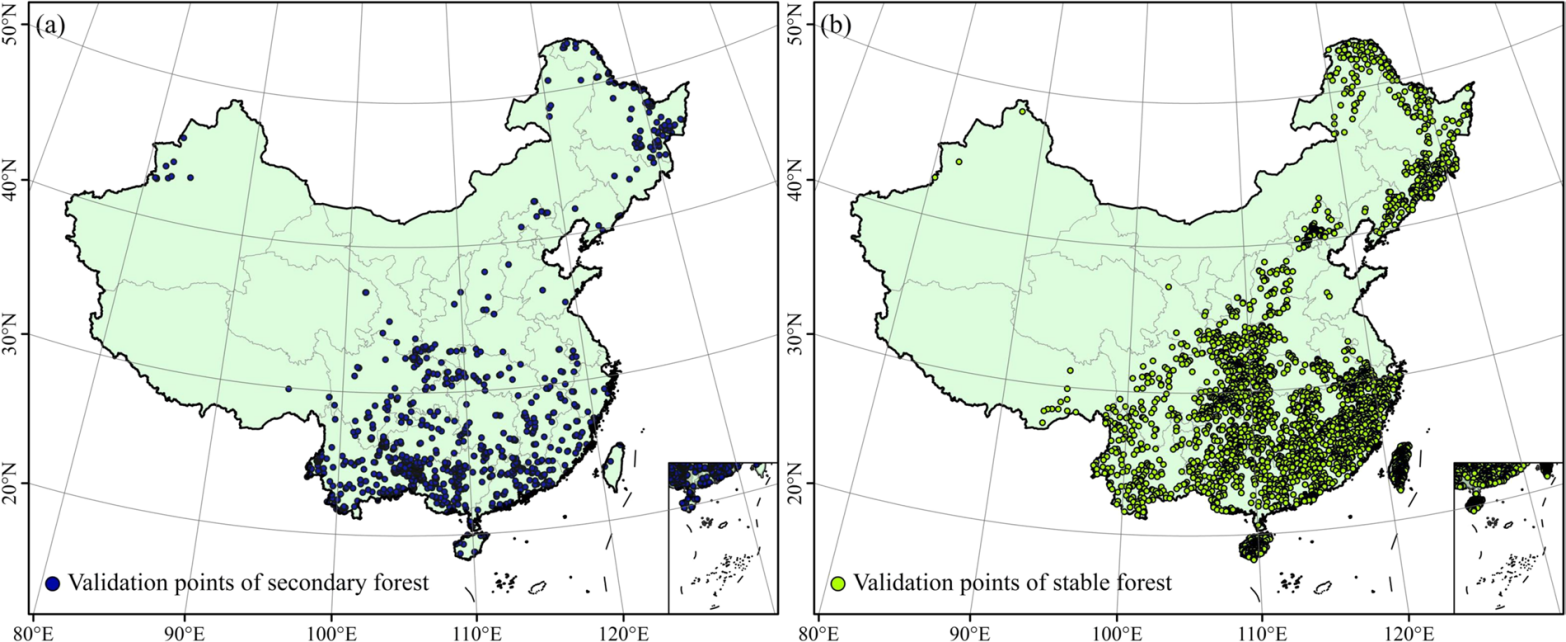
Validation sample points used in the present study. (**a**) secondary forest, (**b**) stable forest.

The 2,072 samples of secondary and 3,000 samples of stable forest, respectively were used to assess the accuracy of the results produced by each algorithm and ensemble^41^ (Fig. 2). Samples for validation were selected randomly from the 7th National Forest Resources Inventory (NFRI) and were compared to the secondary forest maps produced above. The candidate points were visually examined using “Landsat Time Series Explorer”, a shared Application on GEE (https://jstnbraaten.users.earthengine.app/view/landsat-timeseries-explorer). In addition, historical imagery from Google Earth (https://earth.google.com/), GF-6 panchromatic/multispectral (PMS) images (a high-resolution Chinese satellite) (https://data.cresda.cn/#/2dMap) helped to distinguish stable and secondary forest samples. A total of 2,072 validation samples of secondary forest age ranging from 1 to 34 were defined by the re-interpreted approach mentioned above.

Over 3,000 candidates of stable forests were randomly sampled from stable forest maps for validation. The classification of these samples of stable forest was ensured by filtering through many public land cover products. As shown in Table 1, these datasets included AGLC-2000-2015, GLC_FCS, FNF, GLC, CLUD, and GFCC. The categorization of the samples as stable forests was ensured by processing using Python, ArcGIS 10.6, and GEE. The 3,000 samples of the stable forest were then completed after manually removing pixels not following the rules: first, the patch should have pure, intact forest cover and satisfy the definition of forest in the Food and Agriculture Organization of the United Nations (FAO)^42^; second, the point sample of the forest should be located in the center of the forest patch.

**Table 1:** Land cover products used for determining stable forest samples.

Datasets	Resolution	Time period	Source	Reference
AGLC-2000-2015	30 m	2000–2015	https://code.earthengine.google.com/?asset = users/xxc/GLC_2000_2015 [2022-09-01]	^80^
GLC_FCS	30 m	1985/1990/1995/2000/2005/2010/2015/2020	https://data.casearth.cn/thematic/glc_fcs30/95 [2022-09-01]	^81^
The global forest/non-forest map (FNF)	25 m	2007–2018	GEE Collection Snippet: ee.ImageCollection(“JAXA/ALOS/PALSAR/YEARLY/FNF”) [2022-09-1]	^13^
GlobelLand30 (GLC)	30 m	2000/2010/2020	http://www.globallandcover.com/ [2022-09-01]	^36^
China Land Use/Cover Dataset (CLUD)	30 m	1980s/1990/1995/2000/2005/2010/2015	https://www.resdc.cn/ [2022-09-01]	^82,83^
Global Forest Cover Change (GFCC)	30 m	2000/2005/2010/2015	GEE Collection Snippet: ee.ImageCollection(“NASA/MEASURES/GFCC/TC/v3”) [2022-09-01]	^84^

### Detection of establishment times of secondary forest stands

The ages of secondary forest stands were determined by detecting the times of the newest stand establishment using change detection algorithms and Landsat time series data. The present study selected four basic algorithms, namely threshold-based moving average change detection (MACD)^43^, LandTrendr (LT)^44^, VCT, and CCDC algorithms, to detect the establishment times of secondary forest stands. These algorithms were chosen due to their relative advantages in large-scale analysis, performance, convenience, and efficiency, as well as their use in previous studies for estimating the SFAs of specific forests or trees^9,23,43,45–47^. MACD is a thresholding method in which changes are defined as large deviations from the set threshold. The bare soil index (BSI) with a threshold of 0 was used for detecting the stand establishment. LT identifies gradual changes (mainly recovery) in time series by temporal segmentation and linear regression^44,48^. VCT was used to detect the forest regrowth based on the Integrated Forest Z-score (IFZ) threshold^24,25^. CCDC algorithm can fit a curve for each pixel with harmonic model and historical time-series Landsat images and capture changes by comparing model prediction with satellite observation^26^. The Normalized Burn Ratio (NBR) index was widely used to detect the forest dynamic, and it was also used as an input parameter for LT and CCDC. The MACD, LT, VCT, and CCDC were used to identify the establishment times of the secondary forest stands. The establishment time was then converted to forest age in 2020.

#### Data-driven VCT

The VCT suffers various disadvantages, including complex computation, the need for forest samples, and the difficulty of application at a large scale. Therefore, the present study applied a data-driven approach to facilitate the online use of VCT in GEE. The core index, integrated forest z-score (IFZ), was used with VCT to detect the forest dynamic^49^. The IFZ index needed in VCT was calculated as:

1 $$F{Z}_{i}=\frac{{b}_{i}-{\bar{b}}_{i}}{S{D}_{i}},$$

2 $$IFZ=\sqrt{\frac{1}{NB}\mathop{\sum }\limits_{i=1}^{NB}{\left(F{Z}_{i}\right)}^{2}},$$

where the RED, short-wave infrared 1 (SWIR1), and short-wave infrared 1 (SWIR2) bands in Landsat were needed to construct the forest z-score (FZ) and IFZ indices. The ${\bar{b}}_{i}$ and *SD*_*i*_ are the mean and standard deviation of the band *i* spectral values of the forest samples within the image, respectively, and NB is the number of total bands.

Many forest samples were needed to calculate the *b*_*i*_ and *SD*_*i*_ of forest pixels. However, there are differences in structure and spectral properties among different forest types, such as deciduous, mixed forest, open forest, and evergreen forests^50^, as well as among different climate zones, such as temperate, semiarid, and arid zones^24^. Therefore, the use of *b*_*i*_ and *SD*_*i*_ as reference values for the entire study region is inaccurate, particularly at a national scale. In addition, when applying the calculation to many samples, the diversity of forest types hinders the application of the VCT algorithm at a large scale. Secondly, there is a need for a flexible and accurate threshold within the determination of forest recovery. Different IFZ thresholds have been applied among different past studies on forest change. For example, thresholds of 2.5^51^, 3^52,53^, 4.5^54^, 4^55,56^, and 6.5 have been applied in semiarid regions^24^, whereas many other studies do not mention the threshold used^23,57–60^. Although a subtle detail, the IFZ threshold is of importance, particularly when working at a national scale.

**Fig. 3:**
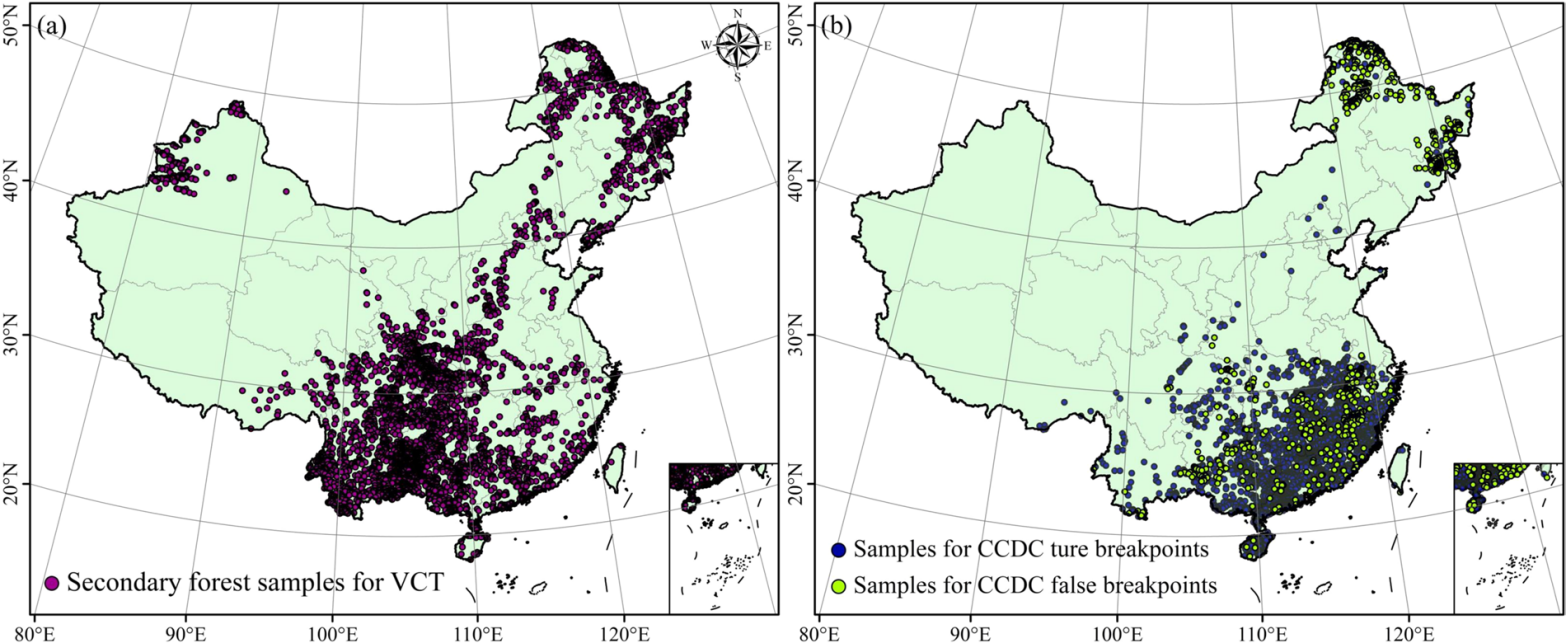
Samples used in the present study. (**a**) for the Vegetation Change Tracker (VCT), (**b**) for the Change Detection and Classification (CCDC).

The current study proposed a data-driven approach for the application of the VCT at a national scale that is more efficient. The steps of the approach are: (1) The samples of stable and secondary forests were filtered based on various conditions, including an area >4,500 m^2^, random selection, and data conversion^61^ (Fig. 3a). (2) A grid with a spatial resolution of 3° was created for the entire study region, thereby overcoming the challenge of mass operation using VCT in the study area. (3) Over 100 forest samples were randomly selected to determine IFZ from the stable forest map produced above. The forest points from the new forest map for 2020 were substituted when no samples in the stable forest map existed in one grid. (4) The samples from the secondary forest map produced above were used to calculate the threshold of IFZ. A pixel was characterized as a forest pixel when the value in the VCT time series was below the threshold for two consecutive years^55^.

#### RF for CCDC

The Random Forest (RF) algorithm for CCDC (CCDC_RF) was developed by introducing the RF model to identify breakpoints of CCDC. The entire CCDC time series was segmented into several shorter time series. These time series were used to produce multiple breakpoints related to changes in land cover. The frequent occurrence of many breakpoints over the entire period at one pixel indicated the strong sensitivity of CCDC for change detection^62^. The false identification of breakpoints by the CCDC was inevitable due to the effects of subtle disturbance, degeneration, and insects on forests at the pixel scale^63^, and not all breaks identified by CCDC represented changes in land cover. The present study aimed to identify breakpoints that only represented afforestation or the transition of non-forest land cover to forest for detecting the establishment of forest stands.

The large quantity of information provided by the breakpoints generated by the CCDC allowed the secondary classification of breakpoints. The coefficients of the fitting model and the variable derived from the three harmonics for each segment were used to classify land cover^64^. The present study aimed to generate a CCDC_RF method in which RF^65^ is used to classify and validate the breakpoints identified by CCDC. The steps used in this process included: (1) All samples were assumed to be correct when they were detected by all four basic algorithms described above at a consistent time (±1 year). False samples were identified as samples for which the results of the algorithms were not consistent with the latest CCDC breakpoints. (2) True and false samples numbering 3,850 and 3,189, respectively were used to train and test the RF for identifying CCDC breakpoints (available at 10.6084/m9.figshare.22224037.v2)^66^ (Fig. 3b). The RF was used to train the classifier model and the latest CCDC breakpoint was used to classify the breakpoints for the entire time series. The maximum number of segments in the CCDC was set to 6, which is sufficient to represent changes in land cover in the time series^67^.

### Ensemble rule

Two rules were used to construct the ensembles. In the first rule, each ensemble was constructed using stacked generalization in which the ahead result was masked from the result of the backward algorithm. For example, the VCT + MACD presents that the MACD’s results were the baseline, and the change results from VCT were then kept in the pixels where MACD detected no changes. This rule was applied to the VCT + MACD, CCDC_RF_OLB + VCT, VCT + CCDC_RF_OLB, and VCT + CCDC_RF_ALB ensembles as well as in VCT + CCDC_RF_ALB + 2 out of 4 (VCR2) ensembles. The ensemble models used in the present study were designed according to the results using individual algorithms for the Detection of establishment times of secondary forest stands. In the remaining 2 out of 4, a forest pixel was needed by at least 2 of the 4 basic algorithms. Within CCDC_RF_OLB, only the latest break was used in the CCDC_RF. In addition, CCDC_RF_ALB was constructed through secondary classification for all breakpoints in the time series.

### Accuracy assessment

**Fig. 4:**
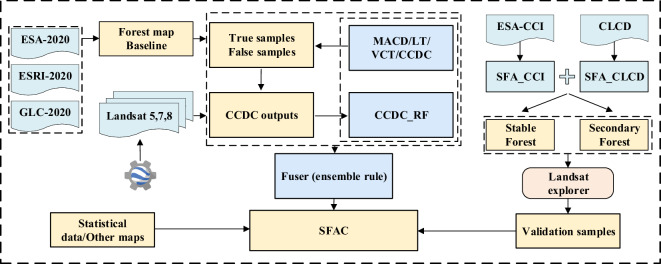
Schematic representation of the process used for mapping secondary forest age in the present study. The colors ‘light cyan’, ‘light yellow’, ‘sky blue’, and ‘light pink’ represent the datasets, methods, tools, and produced data, respectively.

The 2,072 and 3,000 samples of secondary and stable forests, respectively were determined using a labor-intensive exercise. The confusion matrix was obtained based on the validation samples, with the quantitative metrics calculated for each basic algorithm and ensemble, including the overall accuracy (OA), producer’s accuracy (PA), and user’s accuracy (UA). The correct rate (CR) was also used to assess the results of different methods. CR was calculated as the number of correct examples divided by the total detected examples in each class. Figure 4 shows a schematic representing the processes followed in the current study for mapping SFA for China.

#### Comparison with Statistical data

The statistical data of planted forest area and natural forest area was used for comparison with the area of secondary forest and stable forest produced in this study. The statistical afforestation data of China in 2018 was obtained from the China Forest Resources Report (2014–2018) (available at http://www.forestry.gov.cn/). Statistical areas of planted forests would be as the reference data in Chinese provinces with the hypothesis that the planted forest investigated was mainly planted from 1987 to 2018. TW was not included in the provinces investigated in the National Forest Resources Report. In addition, we obtained data on the recovery of forest cover in China for each decade from 1990 to 2020 from FAO^68^. The increase in China’s forest cover from FAO arises from two elements-planted forest area and naturally regenerated forests, consistent with the forest definition detected in this study.

#### Comparison with other maps

The SFA_CCI^69^, SFA_MODIS^70^, SFA_CLCD^71^, and global map of planting years of plantations (GPYP)^72^ were used for comparison with the secondary forest age for China (SFAC)^29^. Since not many SFA products can be compared to SFAC^29^, the SFA data used within the comparison were derived from continuous land cover products, for example, ESA-CCI, MCD12Q1, CLCD(Table 2), and GPYP^72^. The global map of planting years of plantations (GPYP) can be downloaded on the figshare (10.6084/m9.figshare.19070084.v1)^72^ GPYP is in a GeoTIFF format with the 30-meter spatial resolution by recording gridded species types and planting years of global plantations. The Global 1 km forest age datasets (SFA_BGI) can be obtained at 10.17871/ForestAgeBGI.2021^73^. This SFA_BGI provides an ensemble of global estimation of 1 km global forest age in 2010 using forest inventories, biomass, and climate data.

**Table 2:** Land cover datasets of forest age used for inter-comparison.

Datasets	Resolution	Time period	Age period	Source	Reference
European Space Agency Climate Change Initiative-Land Cover (ESA-CCI)	300	1992–2020	1–27	https://climate.esa.int/en/projects/land-cover/ [2022-09-01]	^40^
MODIS Land Cover Type (MCD12Q1)	500	2001–2020	1–18	GEE Collection Snippet: ee.ImageCollection(“MODIS/061/MCD12Q1”) [2022-09-01]	^85^
China Land Cover Dataset (CLCD)	30	1990–2020	1–29	https://zenodo.org/record/4417810#.ZAc1DHZByCg [2022-09-01]	^86^
Global map of planting years of plantations (GPYP)	30	1990–2019	1–34	10.6084/m9.figshare.19070084.v1 [2022-09-01]	^72^
Global 1 km forest age datasets (SFA_BGI)	1000	1990–2010	1–19	10.17871/ForestAgeBGI.2021 [2024-01-01]	^73^

The standard normal error is presented with a 95% confidence interval.

SFA_BGI from 1990 to 2010 was also compiled for comparison with our results. The presented study counted the surface fraction heat plots of the secondary forest according to the age period and at the same spatial resolution of the corresponding reference SFA data in 0.05° spatial grids (Table 2). This approach allowed inconsistencies in spatial and temporal scales to be avoided.

## Data Records

The SFAC dataset produced in the current study can be freely downloaded from figshare (10.6084/m9.figshare.21792557.v2)^29^. The dataset produced in 2022 represents forest age for China in 2020. The data includes 20 files named ‘’sfa_china_2020” with tiff format in a zip. Values from 1 to 34 in the “Age” band represent the age of the forest, where values of 36 and 0 indicate a forest age >34 (not a specific pixel-scale age) and non-forest, respectively. At the same time, the age of 34 to 1 represents the year of forest regrowth ranging from 1987 to 2020. The spatial extent of the dataset includes mainland China and Taiwan but excludes the South China Sea islands. The map is defined in the WGS84 projection and has a 30-m spatial resolution.

The external data used in this paper included the forest map and validation datasets. The new forest base map in 2020 used in our study is available at 10.6084/m9.figshare.22223854.v1^33^. The stable and secondary forest validation samples can also be obtained from 10.6084/m9.figshare.22223911.v1^41^. The stable and secondary forest samples used for the calculation of VCT are available at 10.6084/m9.figshare.22223956.v1^61^. The training and test data for CCDC can be accessed at 10.6084/m9.figshare.22224037.v2^66^.

The three SFA datasets derived from the Moderate Resolution Imaging Spectroradiometer (MODIS), ESA_CCI-LC, and CLCD products for inter-comparison data can be viewed at respectively: (SFA_MODIS: 10.6084/m9.figshare.22225969.v1)^70^, (SFA_CCI: 10.6084/m9.figshare.22225993.v1)^69^, (SFA_CLCD: 10.6084/m9.figshare.22225930.v1)^71^. We provided more access online from GEE in Supplementary DataRecords.

## Technical Validation

### Accuracy assessment

The results of the accuracy assessment showed that using the ensemble method improved accuracies (Table 3). Among the individual algorithms, VCT showed the best performance, with a PA of 72.13%, UA of 49.71%, OA of 71.61%, and mean CR of 75.51%. In contrast, the CCDC achieved the worst results, with a PA of 60.18%, UA of 48.79%, OA of 65.89%, and the lowest mean CR of 63.53%. The LT achieved asymmetric results, with the highest PA of 89.17%, the lowest UA of 20.27%, an OA of 66.42%, and a mean CR of 64.85%. The MACD provided results of intermediate accuracy, with a PA of 57.14%, UA of 63.9%, OA of 65.67%, and mean CR of 80.82%. The UA and PA results of the VCT and CCDC_RF_ALB algorithms were more balanced compared to those for the other single algorithms. The accuracy assessment results for single algorithms suggested that some ensembles were created based on stacked generalization or aggregation.

**Table 3:** The results of the accuracy assessment of each individual and ensemble algorithm.

Scheme	Number of pixels				Accuracy (%)			Correct Rate (%)		
	Correct-secondary forest	Correct-stable forest	Omission	Commission	PA	UA	OA	Secondary forest	Stable forest	Mean
VCT	1030	2602	398	1042	72.13 ± 0.35	49.71 ± 0.90	71.61 ± 0.44	69.50	81.52	75.51
BSI	1324	2007	993	748	57.14 ± 0.80	63.90 ± 0.61	65.67 ± 0.72	70.92	90.73	80.82
LT	420	2949	51	1652	89.17 ± 0.09	20.27 ± 0.18	66.42 ± 0.04	61.76	67.93	64.85
CCDC	1011	2331	669	1061	60.18 ± 0.72	48.79 ± 0.97	65.89 ± 0.45	46.12	80.94	63.53
CCDC_RF_OLB	789	2826	174	1219	81.93 ± 0.11	38.08 ± 0.67	71.27 ± 0.22	67.61	72.37	69.99
CCDC_RF_ALB	853	2805	195	1219	81.39 ± 0.13	41.17 ± 0.76	72.12 ± 0.26	64.18	74.94	69.56
CCDC_RF_ALB + VCT	1259	2367	633	813	66.53 ± 0.52	60.76 ± 0.68	72.49 ± 0.58	71.82	88.16	79.99
VCT + CCDC_RF_ALB	1348	2367	633	724	68.05 ± 0.54	65.06 ± 0.74	73.24 ± 0.56	76.86	88.16	82.50
3 out of 4	273	2986	14	1799	95.12 ± 0.25	13.18 ± 0.02	64.25 ± 0.15	97.85	62.47	80.16
2 out of 4	1033	2766	234	1039	81.53 ± 0.13	49.86 ± 1.0	74.90 ± 0.37	93.32	75.64	84.48
VCT + CCDC_RF_ALB + 2 out of 4 (VCR2)	1370	2363	637	702	68.26 ± 0.49	66.12 ± 0.55	73.60 ± 0.63	76.71	89.20	82.96

The abbreviation of the scheme can be found in the section on the method ensemble rule.

The performances of the ensemble models exceeded those of the individual algorithms. The VCT + CCDC_RF_ALB and CCDC_RF_ALB + VCT ensemble algorithms were constructed in a different order and achieved higher performance than their respective single algorithms. Ensemble algorithm 3 of 4 achieved the highest PA and CR for secondary forests of 95.12% and 97.85%, respectively, and the lowest OA of 13.18%. Ensemble algorithm 2 of 4 produced a PA, UA, OA, and mean CR of 81.53%, 49.86%, 74.90%, and 84.42%, respectively. The proposed ensemble of VCT + CCDC_RF_ALB + 2 of 4 (VCR2) obtained a balanced PA of 68.26%, UA of 66.12%, OA of 73.60%, and mean CR of 82.96% (Table 3). Among the assessed ensemble models, the present study used the superior VCR2 to determine the establishment times of secondary forest stands.

### Comparisons with Statistical data

**Fig. 5:**
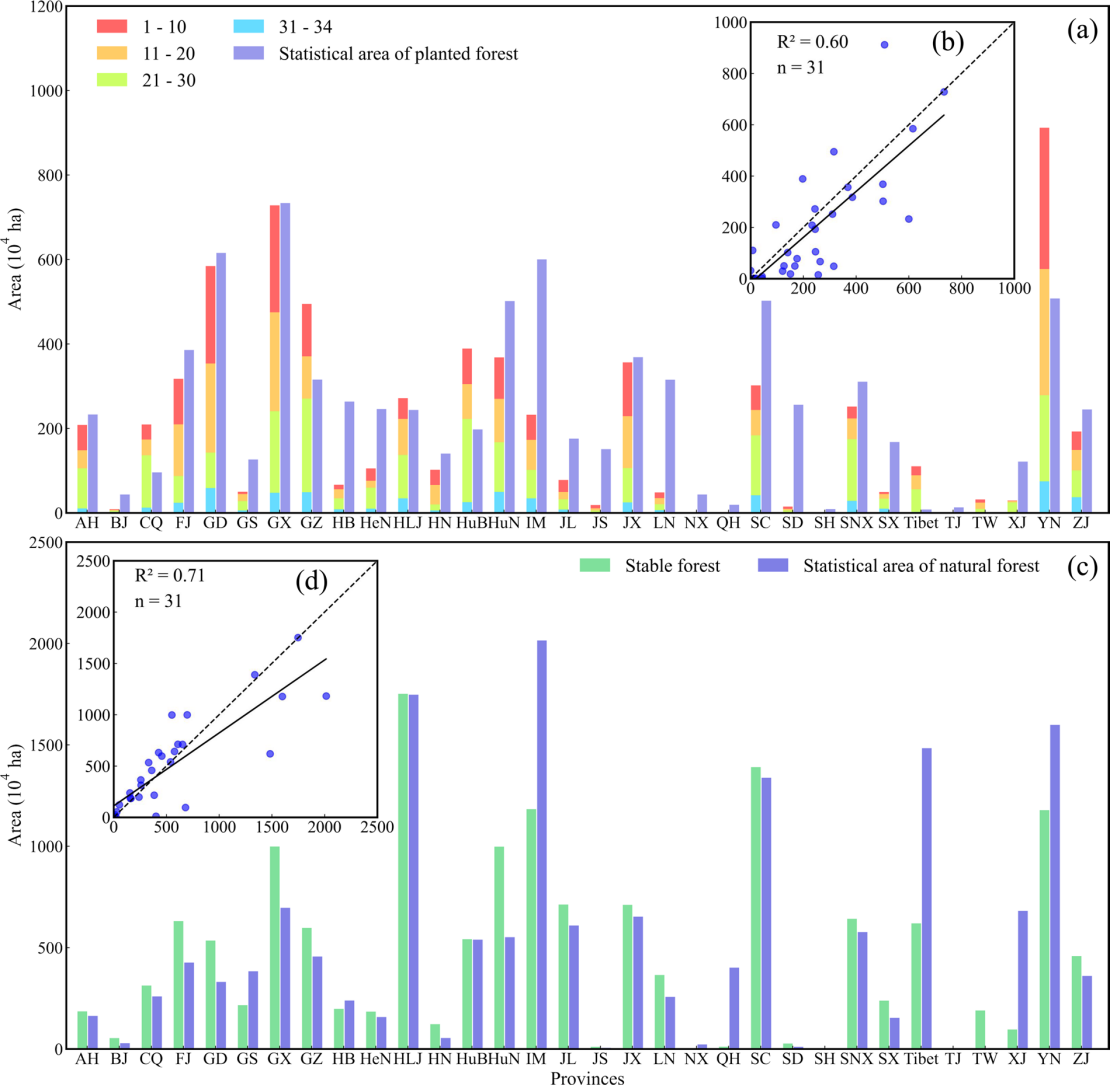
The comparisons with statistical data. (**a**) and (**b**) are histograms and scatter plots of the comparison of the secondary forest area and statistical area of planted forest; (**c**) and (**d**) are histograms and scatter plots of the comparison of the stable forest area and statistical area of natural forest. The codes for provinces are defined in Fig. 1b, n = 31 indicates the TW was not included.

The secondary forest and stable forest produced in this study show a good consistency compared with the statistical data although these data originated from a different standard. There was less difference between the secondary forest area and the statistical area of planted forest in AH, FJ, GD, GX, GX, HLJ, JX, SNX, and ZJ provinces, etc, while a large difference existed in CQ, HB, HeN, IM, LN, SD provinces, etc (Fig. 5a). The secondary forest area with an area of 6.53 × 10^7^ ha had a slight underestimation compared with the statistical area of planted forest with an area of 7.96 × 10^7^ in China. On the other hand, a large difference did not exist in provinces between stable forest area and statistical natural forest area, excepting the HuN, IM, Tibet, and YN provinces (Fig. 5c). The good results were found in the correlations that R^2^ = 0.6 (Fig. 5b), and R^2^ = 0.71 (Fig. 5d), respectively, demonstrating the reliability of our results too. This study indicated that the forest has increased by 2.039 × 10^7^ ha, 1.928 × 10^7^ ha, and 1.978 × 10^7^ ha in 1990–2000, 2000–2010, and 2010–2020, respectively (Table 4, Supplementary Fig. 2). There was only a 5.25% difference in the total area of secondary forest in SFAC from 1990 to 2020 compared to that in FAO (Table 4). Other results, especially the SFA_BGI data, have a big difference compared with the FAO data.

**Table 4:** Estimated area of secondary forest for each period in this study (SFAC)^29^, Food and Agriculture Organization of the United Nations (FAO)^68^, SFA_CCI^69^, SFA_MODIS^70^, and global map of planting years of plantations (GPYP)^72^, SFA_BGI^73^.

Time period	Estimated area in ha (10^7^)						
	FAO	SFAC	ESA_CCI	SFA_MODIS	SFA_CLCD	GPYP	SFA_BGI
1990–2000	1.986	2.039	\	\	1.140	1.403	0.00079
2000–2010	2.361	1.928	0.389	0.373	1.173	0.960	0.00005
2000–2020	1.937	1.987	0.505	0.632	1.698	0.932	\
Total	6.284	5.954	\	\	4.012	3.295	\

### Comparisons with other maps

**Fig. 6:**
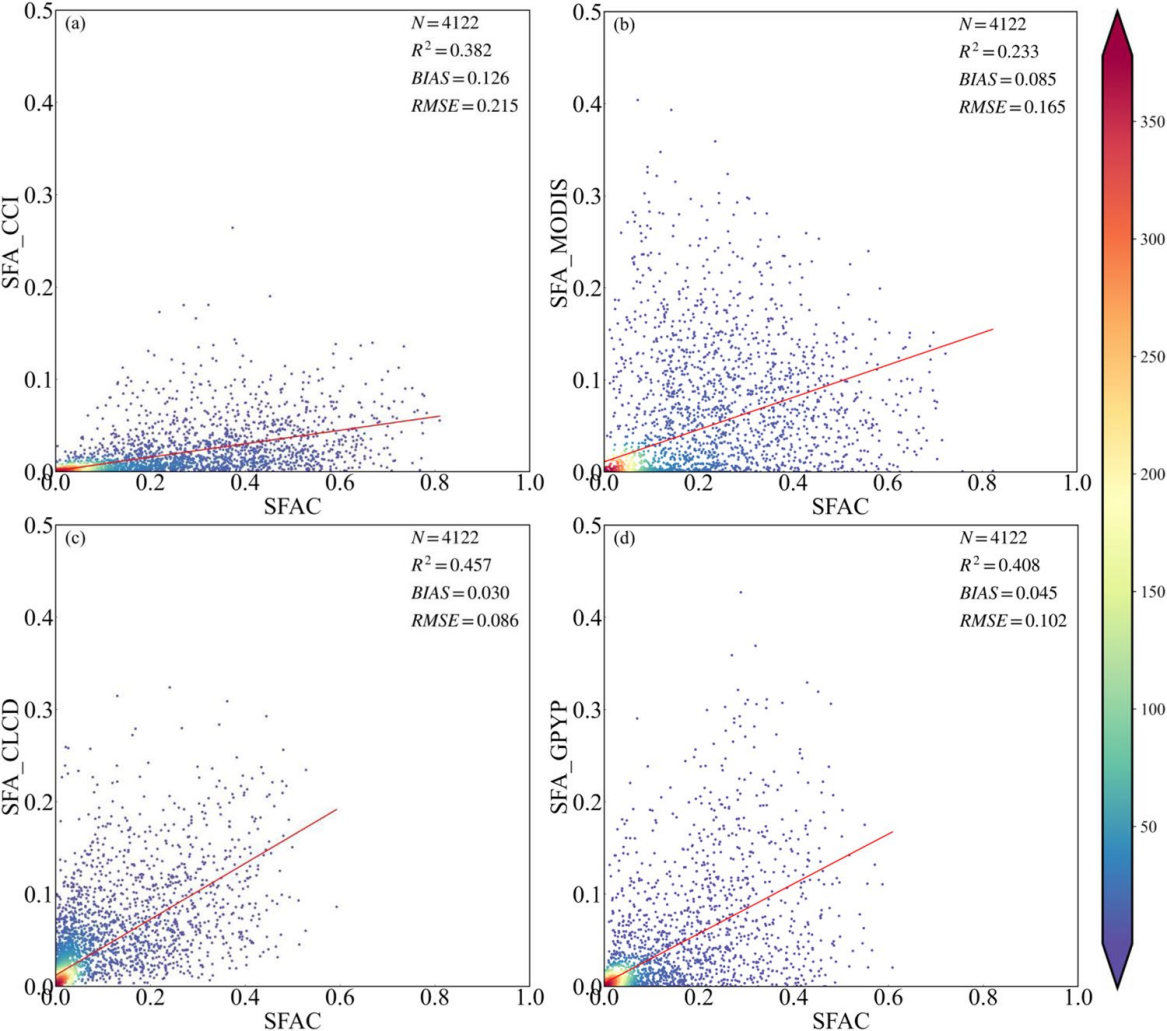
Proportions of secondary forest in a 0.05 ° spatial grid represented as heat plots and showing the relationships between secondary forest age for China (SFAC)^29^ and four reference products at a different resolution. (**a**) Climate Change Initiative (CCI) land cover at 300 m, (**b**) Moderate Resolution Imaging Spectroradiometer (MODIS) at 500 m, (**c**) China Land Cover Dataset (CLCD) at 30 m, (**d**) global map of planting years of plantations (GPYP)^72^ at 30 m.

The maps produced in the current study showed a positive relationship between secondary forest age for China (SFAC)^29^ and reference datasets, although there were some large differences. It should be considered that none of the four inter-comparison products chosen in the present study can be considered reflective of reality as these data were not created specifically for SFA. As shown in Fig. 6, the relationships between SFAC^29^ and SFA_CCI^69^, SFA_MODIS^70^, SFA_CLCD^71^, and GPYP^72^ achieved R^2^ values of 0.382, 0.233, 0.457, and 0.408, respectively. The SFAC^29^ indicated underestimation within all reference SFA data. The comparison between SFAC^29^ and SFA_CLCD^71^ showed higher consistency, whereas that between SFAC^29^ and the two datasets with lower spatial resolutions, SFA_CCI^69^ and SFA_MODIS^70^, showed lower consistency. Some products’ accuracy, spatial resolution, time domain, and methods heavily influenced the low relationships between our results and other derived SFA maps. The results indicated that the non-thematic data heavily underestimated the SFA distribution compared with our results. Our estimated area of secondary forest was closest to the statistics from FAO compared with other SFA data.

**Fig. 7:**
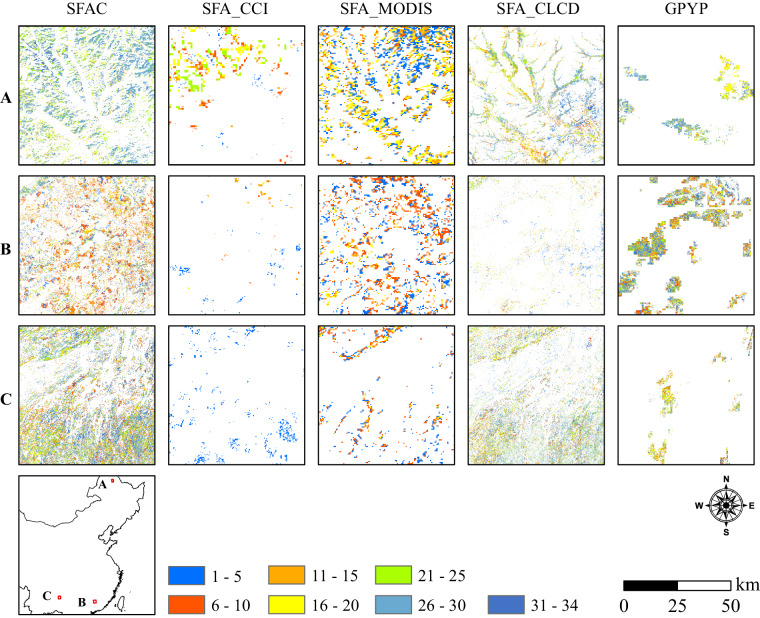
The results of secondary forest age for China (SFAC)^29^, SFA_CCI^69^, SFA_MODIS^70^, and global map of planting years of plantations (GPYP)^72^ for three regions, namely northeast, southeast, and southwest China. The maps only show secondary forests.

The present study further identified spatial differences between these datasets among the three sub-regions presenting dense secondary forests in northeastern, southeastern, and southwestern China (Fig. 7). Various spatial differences were identified when comparing SFAC^29^ to the other SFA maps at a fine spatial scale. Consistency in spatial patterns of secondary forests was identified with the comparison between SFAC^29^ and SFA_MODIS^70^ in regions A and B, as well as between SFAC^29^ and SFA_CLCD^71^ in regions A and C. The SFAC^29^ provided more detailed long-term descriptions of secondary forests at a 30-m resolution compared to that provided by the low-resolution SFA_CCI^69^ and SFA_MODIS^70^. In addition, despite their higher spatial resolution of 30 m and extended age span datasets, SFA_CLCD^71^ and GPYP^72^ underestimated the secondary forest cover. The imperfections in SFA_CCI and SFA_MODIS stem from the low accuracy and spatial resolution of the ESA_CCI and MODIS products. The low comparison consistency in SFA_CLCD and GPYP with SFAC originated from the classification in CLCD and the only used LT algorithm in GPYP. The results showed that the secondary forest identified by SFAC^29^ covered virtually all areas of secondary forest identified in the four reference SFA maps.

## Usage Notes

The national-scale 30-m SFAC^29^ product provides SFA for China over the past 34 years which previous studies have not been able to achieve. This SFAC^29^ dataset can potentially provide information to support forest ecosystem research, including forest biomass, forest carbon sequestration, and forest dynamics.

### The variable thresholds of data-driven VCT

**Fig. 8:**
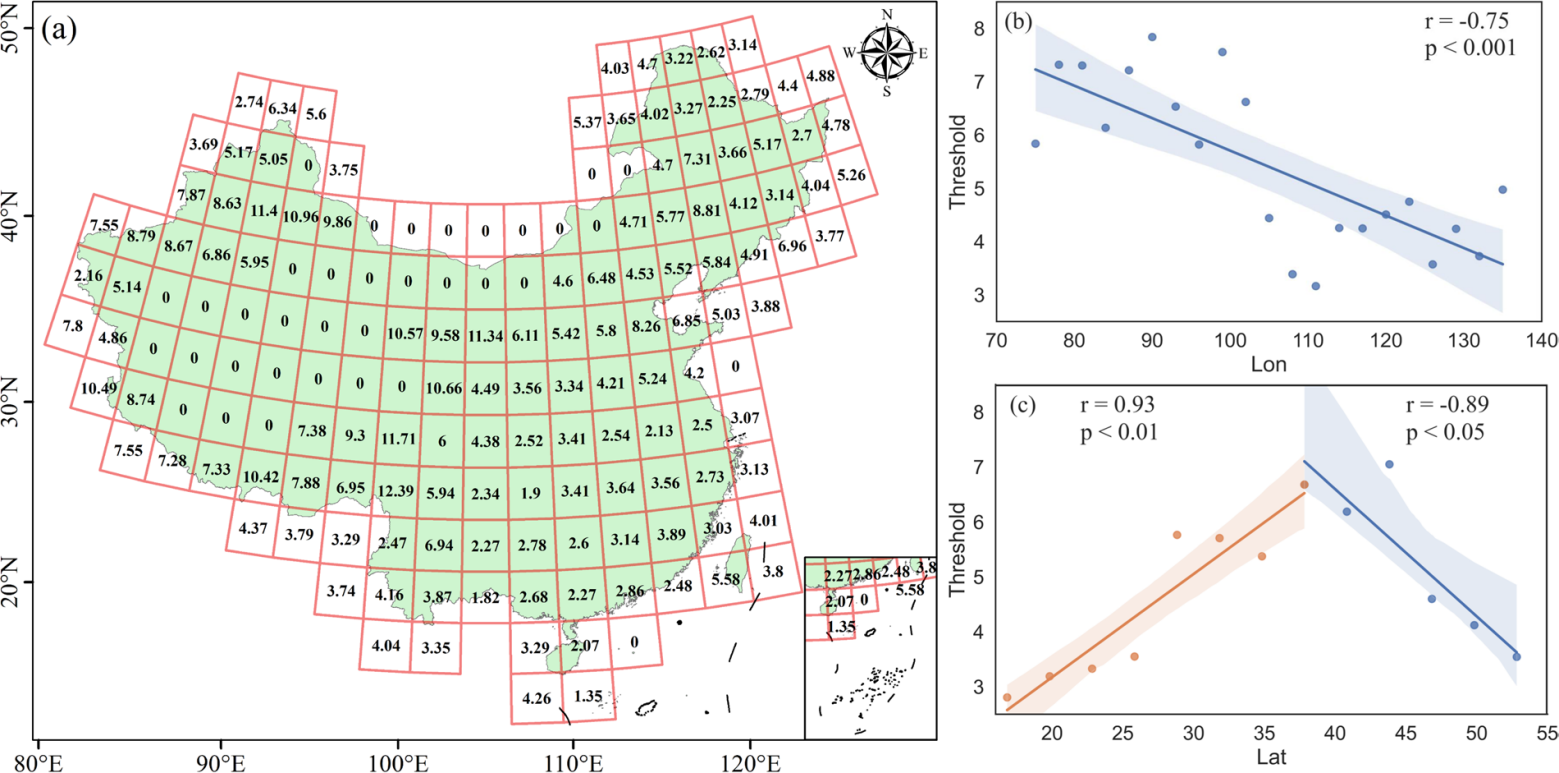
Geographical distribution of the integrated forest Z score thresholds for the Vegetation Change Tracker (VCT). (**a**) at an interval of 3°, (**b**) mean longitudinal IFZ thresholds, (**c**) mean latitudinal IFZ thresholds.

As shown in Fig. 8, the results of the present study showed a heterogeneous IFZ threshold pattern across mainland China and Taiwan. This result could be attributed to region-specific differences in forest ecosystems due to different geographical and climatic conditions. The relatively large and variable IFZ thresholds of Xin Jiang and Tibet could be mainly attributed to their extremely cold climates. The IFZ threshold of western China reached a maximum of 11.4, indicating the establishment of forest stands at a IFZ that was lower than the threshold at the pixel scale. One major reason for the above results is the sparse coverage of unique forest species in the extremely cold regions, such as *Picea asperata* and *Abies fabri*, whereas the lower IFZ threshold in eastern and southern China can be attributed to fast-growing dense forests in the subtropical climate zone.

As expected, the mean IFZ threshold decreased in intervals along the longitude 75° to 135° E, indicating a strong negative correlation (r = −0.75, p < 0.001) (Fig. 8b). However, the trend in mean latitudinal threshold differed from that of the longitudinal threshold. There was an increasing trend in the IFZ threshold along latitude 16.82° to 37.82° N. This result could be attributed to the dense forest cover at low latitudes, with a strong positive correlation (r = 0.93, p < 0.01). On the other hand, Fig. 8c shows a decreasing trend in the IFZ threshold along the latitude 37.82° to 52.82° N with a negative correlation (r = −0.89, p < 0.05). This result can be attributed to the thick forest in the Greater Khingan Mountains, an important forest reserve in northeast China. The variations in the IFZ threshold across the entire study area demonstrated stand establishment of different grades at the growth stage in every interval.

### The performance of the improved CCDC_RF

The CCDC_RF achieved a higher performance in monitoring the establishment of forest stands compared to the CCDC algorithm. The CCDC_RF_OLB achieved an OA, UA, PA, and mean CR of 71.27%, 38.08%, 81.93%, and 69.99%, respectively, exceeding the performance of the single CCDC without the RF model. The CCDC_RF_ALB achieved the best single algorithm performance among the assessed algorithms with an OA of 72.12%, UA of 41.17%, PA of 81.39%, and mean CR of 69.56%. The addition of all breakpoints resulted in obvious improvements in the PA and UA. However, the mean CR achieved by the CCDC_RF_ALB was lower than that of VCT and CCDC_RF_OLB. As shown in Supplementary Fig. 5, the spatial distribution of results produced by the CCDC_RF was similar to that of the original CCDC. However, careful observation revealed that secondary forests shown in Supplementary Fig. 5b had lower coverage than those shown in Supplementary Fig. 5a. This result can be attributed to the retention of true secondary forest due to the removal of false breakpoint information by the RF model.

### Advantages and limitations

The ensemble approach for mapping SFA proposed in the current study produced a distribution of SFAC^29^ that could not be obtained by any single direct or indirect mapping method (Fig. 7). Various past studies have successfully identified the spatial distributions of secondary forest, mature forest, and non-forest land cover using various classification schemes^21,74–76^. While patch size has been shown to be an important indicator influencing classification accuracy^77^, the present study is the first to detect patch size based on a time-series approach^78^, thereby explaining the higher area of secondary forest identified in the present study compared to that in other SFA datasets (land cover from classification). On the other hand, the change detection algorithms showed major differences within their use in a time-series approach due to their basic logic, the density of observation data, and the designed target^19^, thereby limiting their use in detection of forest dynamics^79^. The method proposed in the present study provides an improved forest coverage output compared to that provided by single algorithms.

**Fig. 9:**
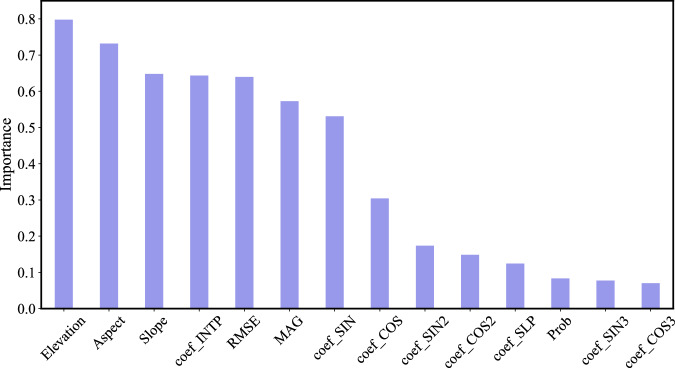
The importance score achieved using the Random Forests model with 14 predictors.

In comparison to common applications using the traditional VCT and CCDC algorithms, the present study developed novel data-driven approaches for VCT and CCDC and applied the RF model to the outputs of the CCDC. The proposed approach showed improved results (Fig. 7, Supplementary Fig. 5). In particular, the results of the CCDC_RF confirmed that it is unreasonable to directly use the outputs of the CCDC for determining secondary forest stands (Table 3). The results of CCDC_RF indicated that it decreased omission and commission errors through application of secondary classification based on many samples defined from the four algorithms. At the same time, the coef_INTP, RMSE, MAG, and coef_SIN variables in outputs of CCDC and topographical factors contributed greatly to the result obtained for CCDC_RF (Fig. 9). CCDC_RF obtained accuracy and Kappa values of 0.98, and 0.96, respectively based on validation against 2,166 samples, higher than the assessment based on validation samples. Theoretically, the CCDC_RF map should provide results that are an improvement over the assessment (Table 3) under the assumption that the whole samples co-defined by the four algorithms for output classification of the CCDC were correct. The discrepancy in assessment can be attributed to errors in the validation samples, despite being manually assessed. Thus, it can be argued that the SFAC^29^ map produced using the optimal ensemble had a higher accuracy with an OA of 73.60% and a mean CR of 82.96% (Table 3).

The PA, UA, and OA from the final ensemble were lower than the accuracy assessment of common classification results. Not only the two types, secondary forests, and stable forests were present, but also the stand establishment time of secondary forests in the temporal domain was determined in this study. Therefore, the CR was also used in this study to show the accuracy of the maps. Actually, the accuracies of secondary and stable forest in the final map were 76.71% and 89.20%. This SFAC product provides a finer description of spatial and temporal domains compared with other maps related to forest age. Overall, our result was better and more reliable now from the validation and comparison based on validation examples, statistical data, and other products. However, the age estimation of the old forest needs further exploration in future work.

### Supplementary information

Supplemental Information

## Data Availability

The codes used in data generation and processing are in two formats, JavaScript used in GEE and Python. The codes are available in GitHub at: (https://github.com/Zhangshaoy/SFAC.git). Each repository includes a guide for the use of the codes. An online visualization map using the GEE experimental app is also provided: (https://zsy11600.users.earthengine.app/view/sfac).
