# Supplementary material for: Mapping of secondary forest age in China using stacked generalization and Landsat time series
Source: Sci Data. 2024 Mar 16;11:302. doi: 10.1038/s41597-024-03133-2 (PMC10944476; doi:10.1038/s41597-024-03133-2)
Supplement: Supplementary file 1 — Supplemental Information [file 41597_2024_3133_MOESM1_ESM.pdf]

Supplemental Information

Mapping of secondary forest age in China using stacked generalization and Landsat time series

Shaoyu Zhang[1], Hanzeyu Xu[1,2], Aixia Liu[3], Shuhua Qi[1], Bisong Hu[1], Min Huang[1], Jin Luo

Supplemental Information 1
Results Analysis..3
DataRecords...6

Results Analysis

The spatiotemporal patterns of SFAC.

Fig. S1a shows the spatial distribution of secondary forest age for China (SFAC) produced using the proposed VCR2 scheme. Overall, the estimated stand ages of secondary forests (stand age ≤ 34) in China had a mean and median of 25 and 18 years, respectively. Southeast and northeast China showed higher coverages of secondary forests compared to the north and west. The area of secondary forest in China represented in the SFAC of 6.53×10^7 ha accounted for 30.13% of the total forest area in China. Fig. S1b shows the spatial distribution of stable forests (stand age ≥ 35) within the SFAC. North-east and central China showed the highest coverages of stable forest.

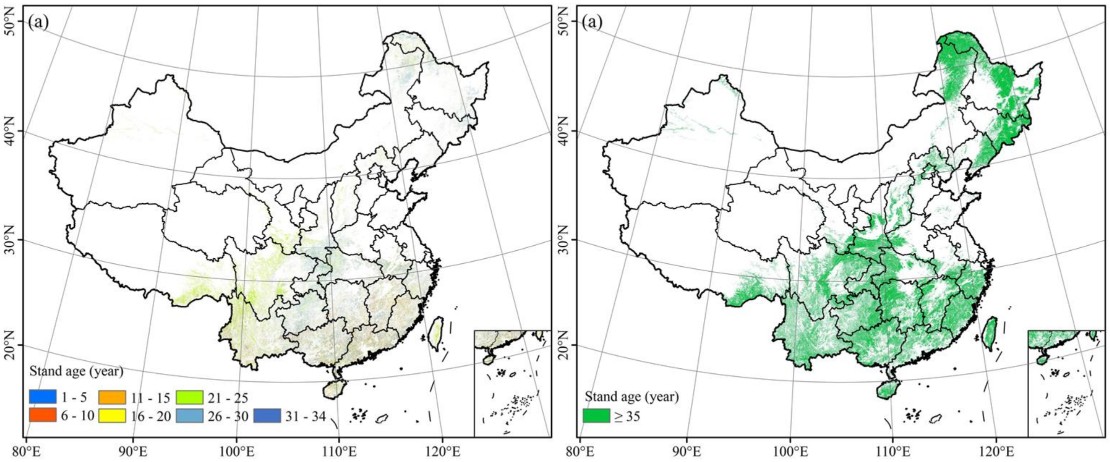

Fig. S1 The spatial distribution of forest age in China for 2021 at a 30-m resolution; (a) the distribution of secondary forest age (b), the distribution of stable forests (age ≥ 35 years).

There were no major differences in the coverage of secondary forests among the different years except for forests with an age of 28 years (Fig. S2). The mean area of secondary forest in every age group was 0.19×10^7 ha. Secondary forests with an age of 28 years showed a wide coverage of 5.21×10^7 ha. Secondary forest with an age of 1 year showed a relatively small coverage of 0.083×10^7 ha. It is possible that these younger forests were not fully detected due to the limited duration of growth prior to 2022. More than 80% of secondary forests were aged between 1 to 28 years, indicating relatively young secondary forest in China.

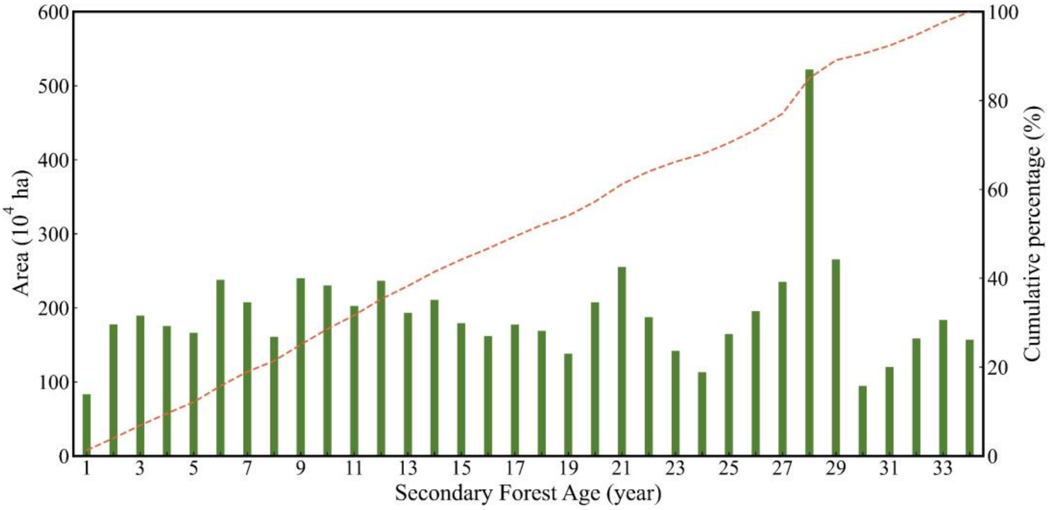

Fig. S2 Areas of secondary forest within the secondary forest age for China (SFAC) dataset (age ≤ 34) among different ages. The dashed orange line represents the cumulative percentage of secondary forests.

As shown in Fig. S3, the coverages of secondary forest were widely different among the different provinces of China, which could be attributed to variable geographical conditions. Yunnan province showed the most secondary forest (0.91×10^7 ha), followed by the provinces of Guangxi (0.73×10^7 ha), Guangdong (0.58×10^7 ha), and Guizhou (0.19×10^7 ha). There were relatively small areas of secondary forests in Tianjin province (0.00051×10^7 ha), followed by the provinces of Ningxia (0.00068×10^7 ha), Shanghai (0.0013×10^7 ha), and Beijing (0.00862×10^7 ha). The study region had an area of secondary forest of 0.20×10^7 ha. As expected, Heilongjiang province had the largest area of stable forest (1.75×10^7 ha), followed by the provinces of Sichuan (1.39×10^7 ha), Inner Mongolia ($1,18 \times 10^7$ ha), and Yunnan ($1,18 \times 10^7$ ha). Shanghai showed the lowest coverage of stable forest (0.00008×10^7 ha), followed by the Tianjin (0.0017×10^7 ha), Ningxia (0.0025×10^7 ha), and Jiangsu (0.010×10^7 ha), with all these regions located in the developed areas and eastern China. The mean provincial area of stable forest in China was 0.47×10^7 ha. The area of secondary forest was greater than half of the area of stable forest in 60% of the provinces. The provinces of Anhui, Guangdong, Jiangsu, and Shanghai had areas of secondary forests that exceeded that of stable forests, indicating the effects of large afforestation activities over the last 34 years.

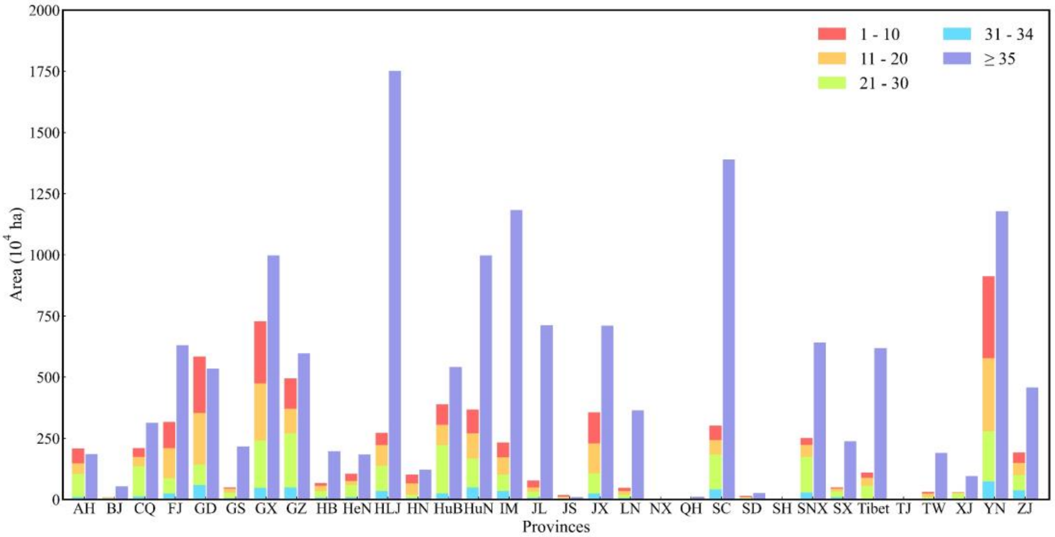

Fig. S3 Areas and ages of secondary and stable forest in China among different provinces.

The cumulative area of secondary forests for eight shelterbelt programs accounted for 46.03% (3.0×10^7 ha) of the total area of secondary forests in China. The secondary forests attributable to shelterbelt programs were mainly concentrated in the upper and middle reaches of the Yangtze River (1.90×10^7 ha) (Fig. S4a, b). Among the shelterbelt areas, that for the Liaohe River showed the lowest area of secondary forest of 0.0068×10^7 ha. Despite the large cumulative area of the three shelterbelt regions in the north, forests in these areas accounted for 13.91% (0.078×10^7 ha) of total forest area in this region due to the extreme cold climate. The HT region showed a higher secondary forest: stable forest ratio than that of the other shelterbelt programs. TH showed the oldest secondary forest age (median and mean ages of 26 and 25 years, respectively), followed by the Yangtze River (median and mean ages of 26 and 22 years, respectively), the Yellow River (median and mean ages of 25 and 23 years, respectively), TN (median and mean ages of 24 and 21 years, respectively), the Pearl River (median and mean ages of 24 and 21 years, respectively), LH (median and mean ages of 20 and 20 years, respectively), the coastal region (median and mean ages of 20 and 20 years, respectively), and HT (median and mean ages of 20 and 18 years, respectively).

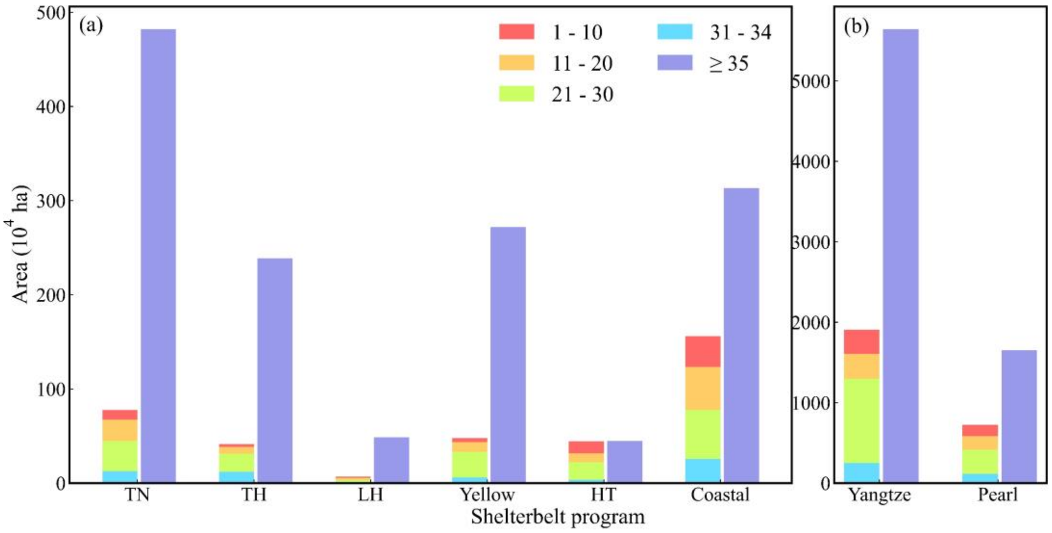

Fig. S4 The areas of secondary and stable forest among different shelterbelt programs and ages.

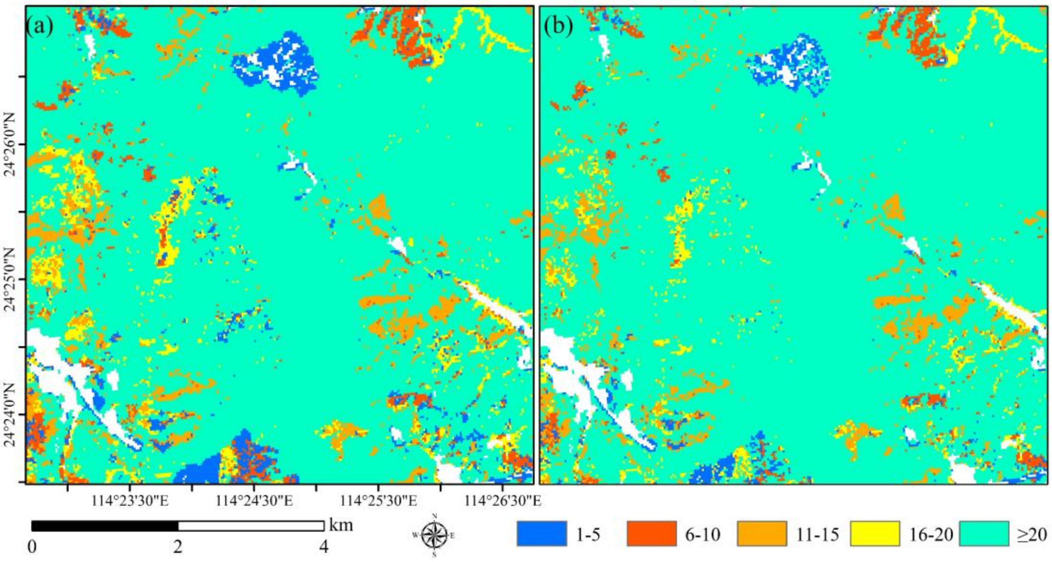

Fig. S5 The results of the Change Detection and Classification (CCDC); (a) without the Random Forest (RF) model, (b) with the RF model (CCDC_RF_OLB).

DataRecords

The reference forest map for 2020 produced by the current study is available as an Earth Engine Image Collection and can be downloaded at: (https://code.earthengine.google.com/?asset=projects/my-image-assets/assets/China/Forest_2020_china_v2).

The stable and secondary forest validation samples can also be obtained from GEE (https://code.earthengine.google.com/?asset=projects/my-image-assets/assets/China/ShareData/ValidationSamples).

The stable and secondary forest samples used for calculation of VCT are available from: (https://code.earthengine.google.com/?asset=projects/my-image-assets/assets/China/Cci_points).

The training and test data for CCDC can be accessed at: (https://code.earthengine.google.com/?asset=projects/my-image-assets/assets/China/CCDC/ccdc_use_training_data_third).

The three SFA datasets derived from the Moderate Resolution Imaging Spectroradiometer (MODIS), CCI, and CLCD products for inter-comparison data can be viewed in GEE: (SFA_MODIS: https://code.earthengine.google.com/?asset=projects/my-image-assets/assets/China/SFA_OtherData/sfa_modis, SFA_CCI: https://code.earthengine.google.com/?asset=projects/my-image-assets/assets/China/SFA_OtherData/sfa_cci_china, SFA_CLCD: https://code.earthengine.google.com/?asset=projects/my-image-assets/assets/China/SFA_OtherData/China_secondary_age_land_clcd).

An online visualization map of SFAC using the GEE experimental app is also provided: (https://zsy11600.users.earthengine.app/view/sfac).
